# X-Linked Hypophosphatemia: Role of Fibroblast Growth Factor 23 on Human Skeletal Muscle-Derived Cells

**DOI:** 10.1007/s00223-025-01415-4

**Published:** 2025-09-09

**Authors:** I. Falsetti, G. Palmini, S. Donati, C. Aurilia, R. Zonefrati, L. Di Filippo, A. Giustina, S. Giannini, G. P. Arcidiacono, T. Iantomasi, D. Lazzerini, P. Joos-Vandewalle, C. Lee, M. L. Brandi

**Affiliations:** 1 Department of Experimental and Clinical Biomedical Sciences “Mario Serio”, University of Florence, Viale Pieraccini 6, 50139 Florence, Italy; 2 FirmoLab, Fondazione F.I.R.M.O. Onlus and Stabilimento Chimico Farmaceutico Militare (SCFM), 50141 Florence, Italy; 3 Institute of Endocrine and Metabolic Sciences, San Raffaele Vita-Salute University and IRCCS Hospital, 20132 Milan, Italy; 4 Clinica Medica 1, Department of Medicine, European Reference Network on Rare Bone Diseases (ERN BOND), University of Padua, 35128 Padua, Italy; 5 Medical Affairs, Kyowa Kirin, Milan, Italy

**Keywords:** X-linked hypophosphatemia, Muscle satellite cells, FGF23, Myogenesis

## Abstract

X-linked hypophosphatemia (XLH) is a rare and progressive disease, due to inactivating mutations in the phosphate-regulating endopeptidase homolog X-linked (*PHEX*) gene. These pathogenic variants result in elevated circulating levels of fibroblast growth factor 23 (FGF23), responsible for the main clinical manifestations of XLH, such as hypophosphatemia, skeletal deformities, and mineralization defects. However, XLH also involves muscular disorders (muscle weakness, pain, reduced muscle density, peak strength, and power). Although XLH is characterized by muscle disorders, to date there are few studies on the action of FGF23 on muscle. Therefore, this study aims to evaluate the effects of FGF23 in an in vitro model of skeletal muscle satellite cells derived from human biopsies (hSMCs). After isolating and characterizing three lines of hSMCs from three volunteers, we evaluated the effect of FGF23 on the proliferative and myogenic differentiation process. We observed that none of the three concentrations of FGF23 tested (1, 10, 100 ng/mL) affected the proliferative process after 48 h of treatment. On the contrary, after 24 and 48 h of treatment, FGF23 resulted in a significant reduction in the gene expression of the myogenic regulatory factors family (Myf-5, MyoD-1, Myogenin, and MRF4), irisin, myosin heavy chain, myostatin, desmin, FGF23 receptors (FGRF1-4) and KLOTHO coreceptor. We, therefore, hypothesized that FGF23 is directly involved in the muscular disorders that characterize XLH, and clarifying these effects at the molecular and cellular level is essential to elucidate XLH pathogenesis and, consequently, its management.

## Introduction

X-linked hypophosphatemia (XLH; Online Mendelian Inheritance in Man (OMIN) #307800) is a genetic, musculoskeletal, progressive rare disease with an incidence of 3.9 per 100,000 live births [1]. It is due to inactivating mutations in the phosphate-regulating endopeptidase homolog X-linked (*PHEX*) gene (OMIM: #300550) and follows an X-dominant heritance transmission [2].

The *PHEX* gene is located on chromosome Xp22.1 and is expressed mainly in osteoblasts and osteocytes. It encodes for an enzyme that degrades local small integrin-binding ligands, N-linked glycoproteins (SIBLING proteins), and is responsible for decreased levels of fibroblast growth factor 23 (FGF23) [3]. To date, more than 850 disease-causing variants in the *PHEX* gene have been identified in XLH patients, resulting in high circulating levels of FGF23 [4].

FGF23 is a hormone belonging to the endocrine FGF family, which is capable of exerting a biological effect even far from the site of secretion via the bloodstream [5]. Specifically, FGF23 is synthesized by osteocytes and osteoblasts and regulates phosphate homeostasis [6]; it suppresses phosphate reabsorption by down-regulating the expression of the sodium-phosphate co-transporter (NaPi-2a and NaPi-2c) in the proximal renal tubules, and it controls phosphate homeostasis through inhibition of renal 1-α-hydroxylase enzyme (resulting in reduced levels of active vitamin D) and parathyroid hormone secretion [7].

FGF23 activity depends on tyrosine kinase FGF receptors (FGFRs). Four isoforms of FGFRs have been identified (FGFR1, FGFR2, FGFR3, FGFR4) [5]. After ligand binding, the presence of α-KLOTHO, a type I membrane protein, is required. α-KLOTHO functions as a coreceptor and has been shown to increase FGF23’s affinity for its FGFRs 20-fold, stabilize the FGF23-FGFRs complex and ensure their activation [8, 9].

Excess of FGF23 induces hypophosphatemic diseases and among these XLH is the most prevalent form of genetic FGF23-related hypophosphatemic rickets (~ 80% of all cases of hypophosphatemic rickets) [10, 11]. In particular, the clinical and biomedical characteristics of XLH are largely delineated in bone tissue, as high FGF23 levels lead to renal phosphate loss, hypophosphatemia, skeletal deformities, and mineralization defects [12].

The main clinical manifestations of XLH in children are rickets, short stature, pain, and deformities in the lower limbs resulting in difficulty walking [13, 14]. Other features are cranial abnormalities, dental abscesses and caries [14]. In adults, in addition to the symptoms of early childhood, there are those due to the progressive worsening of the disease, such as osteomalacia, fragility fractures, bone pain, early-onset osteoarthritis, and enthesopathy [15]. This leads to reduced mobility and a deterioration in the health-related quality of life of these patients [16].

However, XLH also affects muscle function, causing muscle weakness, pain, reduced muscle density, peak strength and power, changes in muscle composition, alterations of transmembrane potential, creatinuria, and rhabdomyolysis [3, 17–19]. It has been demonstrated that the structural and functional deficits in the skeletal muscles of these patients are caused by chronic hypophosphataemia [20–23].

Despite the muscular symptoms that XLH causes, very few reports are available about the effect of FGF23 on skeletal muscle tissue. Therefore, further studies, including in vitro analysis at the molecular and cellular level are necessary for a better and more specific understanding of how FGF23 acts not only on the regulation of the bone cells but also on skeletal muscle cells. Since bone and muscles are in crosstalking, the study of the effects of FGF23 on the muscle compartment could open new strategies to understand the interactions between these two important tissues.

In relation to this, the aim of our study was to evaluate the effect of FGF23, tested at different concentrations, both on proliferation and on myogenic differentiation of human skeletal muscle satellite cells (hSMCs).

## Materials and Methods

### Isolation of hSMCs and Cultures

hSMCs were obtained from biopsies of three healthy adult volunteers after having signed an informed consent in accordance with a protocol approved by the Local Ethics Committee of the AOU Careggi, Florence (Italy), for human studies (Ref. BIO 14.017), as well as with the ethical standards outlined in the Declaration of Helsinki (1964) and its subsequent amendments or comparable ethical standards.

Biopsies, within 24 h of collection, were processed in the laboratory in order to obtain hSMCs, as previously reported [24]. hSMCs were cultured in the Skeletal Muscle Cell Growth Medium (GM) (Promocell GmbH, Heidelberg, Germany), containing a low concentration of phosphate.

In order to increase adhesiveness and maintain the phenotype, plates coated with Matrigel^®^ (BD Company, Franklin Lakes, NJ, USA, 354234) were used in all performed experiments.

### Osteogenic, Adipogenic, and Myogenic Differentiation

In order to confirm the stem cell phenotype of our established lines, hSMCs were induced to differentiate toward the osteogenic, adipogenic, and myogenic phenotypes with specific culture media, which were refreshed twice a week.

Osteogenic differentiation was assessed by qualitative evaluation of the production of hydroxyapatite (HA) deposits by cytochemical staining after 21 days of induction. To perform osteogenic differentiation assay, hSMCs were plated in 24-well plates in GM and upon reaching 80/90% confluence induced to differentiate toward the osteogenic phenotype with the osteogenic medium (OM), consisting of Ham’s F12 Coon’s modification medium supplemented with 10% Fetal Bovine Serum (FBS), 100 IU/mL penicillin, 100 μg/mL streptomycin (control medium, CM) with 10 nM dexamethasone, 0.2 mM sodium L-ascorbyl-2-phosphate, 10 mM β-glycerol-phosphate, and 1 μg/mL calcein. After 21 days of induction, hSMCs were fixed with 4% paraformaldehyde for 10 min and washed twice with Dulbecco’s phosphate buffered saline (DPBS) and three times with ultrapure H_2_O. The qualitative evaluation of the presence of HA deposits was carried out using epifluorescence microscopy (Zeiss). HA deposits can be easily detected by epifluorescence thanks to the presence of calcein in the OM. HA deposits were stained in fluorescent red, while the nuclei were counterstained with propidium iodide for 5 min (diluted 1:100 in DPBS) and stained in fluorescent green.

Adipogenic differentiation was assessed by monitoring the production of lipid intracellular vesicles by cytochemical staining after 14 days of induction. The hSMCs were plated in 24-well plates in GM and upon reaching 80/90% confluence induced to differentiate toward the adipogenic phenotype using the specific adipogenic medium (AM), consisting of CM supplemented with 1 µM dexamethasone, 1 µM bovine insulin, 0.5 mM Isobutylmethylxanthine, and 100 µM indomethacin. After 14 days of induction, evaluation of the adipogenic phenotype was performed on hSMCs by cytochemical staining using Oil Red O solution. The presence of lipid vesicles is confirmed by observation in bright field with laser scanning confocal microscopy (LSCM-900, Zeiss).

Myogenic differentiation was confirmed by immunofluorescent staining to evaluate the presence of myosin heavy chain (MHC) after 10 days of induction. The hSMCs were plated in 24-well plates in GM medium and upon reaching 70% confluence were induced to differentiate toward the myogenic phenotype using Skeletal Muscle Cell Differentiation medium (DM) (PromoCell GmbH, Heidelberg, Germany). After 10 days of induction, the cells were fixed with 4% paraformaldehyde for 10 min, then washed with DPBS (twice) and ultrapure H_2_O (three times), and MHC immunofluorescence was performed.

### Immunofluorescence

Immunofluorescence staining was performed in hSMCs after myogenic induction to highlight the presence of MHC. The hSMCs, seeded in 24-well plates, were fixed with 4% paraformaldehyde for 10 min after 10 days of myogenic induction, washed twice with DPBS, three times with ultrapure H_2_O, and permeabilized with Triton 0.1% X-100 for 30 min at 37 °C in a humidified atmosphere with 5% CO_2_. After three washes with DPBS, hSMCs were incubated for 30 min with RNAse in 2% bovine serum albumin in DPBS at 37 °C in a humidified atmosphere with 5% CO_2_. Then, hSMCs were washed three times with DPBS and incubated overnight at 4 °C with the primary antibody for MHC (Abcam, Cambridge, UK). The following day, after removal of the primary antibody and three washes with DPBS and three washes with 2% bovine serum albumin in DPBS, hSMCs were incubated for 45 min in the dark at room temperature with the secondary antibody SuperClonal goat anti-mouse IgG (H + L), Alexa Fluor 488 conjugate (Thermo Fischer Scientific, Waltham, MA, USA). After three washes with DPBS, the nuclei were stained with propidium iodide, diluted 1:100 in DPBS, for 5 min at room temperature in the dark. Observation of hSMCs was performed by LSCM-900 (Zeiss).

### FGF23 Treatment

In our experiments, three different concentrations (1, 10, and 100 ng/mL) of FGF23 (Sigma-Aldrich, Saint Luis, MO, USA, #SRP3039) were tested and added to the DM.

### Cell Proliferation Assay

The proliferation assay was performed on hSMCs treated with three different concentrations of FGF23, using the bromodeoxyuridine (BrdU) incorporation ELISA kit (Abcam, Cambridge, UK, ab126556). The hSMCs were seeded in 96-well plates at a density of 1 × 10^4^ cells/well. The following day, serum-free medium was added to the hSMCs for 24 h. Then, the cells were treated with FGF23 for 24 h and the following day were incubated with BrdU for a further 24 h. At the end of the incorporation time, hSMCs were collected according to the kit instructions. The results obtained are reported as a percentage of the respective absorbance measured in the control group.

### RNA Extraction and Gene Expression Analysis by Real-Time Quantitative Reverse Transcription Polymerase Chain Reaction

Gene expression analysis was performed in hSMCs in DM after 24 and 48 h of treatment with FGF23. The total RNA of hSMCs was extracted using Qiazol Lysis Reagent (Invitrogen, USA), as previously reported [25]. 500 ng of the isolated RNA was reverse transcribed with the QuantiTect Reverse Transcription kit (Qiagen). RPS18 was used as an internal control. Quantitative real-time PCR (qPCR) was conducted using TaqMan assay on a Rotor-Gene Q real-time PCR cycler (QIAGEN). From the cDNA samples, standard curves were constructed for quantitative analysis using serial dilutions. All points for standard curves and unknown samples were performed in triplicate, and data were expressed as means ± SD of the number of mRNA molecules. The primer sequences used for amplification of all the genes described above are listed in Table 1.

**Table 1:** A list of primers and TaqMan probe sequences, with the amplicon size and annealing temperature used for the experiments

Gene	Primer sequences (5ʹ-3ʹ) and TaqMan probes	Amplicon size (bp)	T_m_ (°C)
MyoD-1 for	GACGTGCCTTCTGAGTCG		
MyoD-1 probe	6-FAM/CGCTGCTCT/Zen/CTCCCTCGCTG/3IABkFQ	148	55
MyoD-1 rev	CTCAGAGCACCTGGTATATCG		
Myf-5 for	ATGCCATCCGCTACATCG		
Myf-5 probe	6-FAM/CCCCACCTC/Zen/CAACTGCTCTGAT/3IABkFQ	145	55
Myf-5 rev	ACAGGACTGTTACATTCGGC		
MRF4 for	CCCTGGAATGATCGGAAACA		
MRF4 probe	6-FAM/ATCTTGAGG/ZEN/GTGCGGATTTCCTGC/3IABkFQ	95	55
MRF4 rev	CTTCAGCTACAGACCCAAACA		
Myogenin for	AGCGAATGCAGCTCTCAC		
Myogenin probe	6-FAM/TGACCCTAC/Zen/AGATGCCCACAACC/3IABkFQ	150	55
Myogenin rev	TGTGATGCTGTCCACGATG		
Desmin for	AACGCGATCTCCTCGTTG		
Desmin probe	6-FAM/CAATTCTGC/ZEN/GCTCCAGGTCAATGC/3IABkFQ	101	55
Desmin rev	GAGAACAATTTGGCTGCCTTC		
Irisin for	ACTATGTACTCCGTATCCTCCTC		
Irisin probe	6-FAM/CCAGCAGAA/ZEN/GAAGGATGTGTCGGAT/3IABkFQ	126	55
Irisin rev	TGTCATCGGATTTGCCATCT		
Myostatin for	GCTCTTTGGAAGATGACGATTAT		
Myostatin probe	ACCATGCCTACAGAGTGTAAGTAGTCCT	90	60
Myostatin rev	TTCCATCCACTTGCATTAGAAA		
MHC for	GAGTCCTTTGTGAAAGCAACAG		
MHC probe	6-FAM/CAAGTCTTC/Zen/CCCATGAACCCTCCC/3IABkFQ	143	55
MHC rev	GCCATGTCCTCGATCTTGTC		
FGFR1 for	GCTTCACTTAAGAAATGTCTCCTT		
FGFR1 probe	56-FAM/TGGCGGGTA/ZEN/ACTCTATCGGACTCT/3 IABkFQ	106	58
FGFR1 rev	TTCCAGAACGGTCAACCA		
FGFR2 for	GGATAACAACACGCCTCTCTT		
FGFR2 probe	56-FAM/TCCGAGTAT/Zen/GAACTTCCAGAGGACCC/3IABkFQ	119	60
FGFR2 rev	CTTGCCCAGTGTCAGCTTAT		
FGFR3 for	GAAGAACGGCAGGGAGTT		
FGFR3 probe	56-FAM/ATCAGCAGT/Zen/GGAGCCTGGTCATG/3IABkFQ	124	60
FGFR3 rev	CACGACGCAGGTGTAGTT		
FGFR4 for	TGAGAGCTGTGAGAAGGAGAT		
FGFR4 probe	56-FAM/TCTTGTCCC/Zen/TGGAGGCCTCTGA/3IABkFQ	115	58
FGFR4 rev	TGTGGCAAGCTCCACTTC		
KLOTHO for	ACCAGCTGAGGGTGTATTATATG		
KLOTHO probe	56-FAM/ACGAAGCTC/Zen/TCAAAGCCCACATACTG/3IABkFQ	116	60
KLOTHO rev	AGCTGTGCGGTCGTTAAA		
RPS18 for	GATGGCAAAGGCTATTTTCCG		
RPS18 probe	6-FAM/TTCAGGGAT/ZEN/CACTAGAGACATGGCTGC/3IABkFQ	132	60
RPS18 rev	TCTTCCACAGGAGGCCTAC		

*Bp* base pairs of amplicon size, *Ta* annealing temperature

### Statistical Analysis

Each experiment was performed in triplicate and repeated three times. Data are reported as mean ± SD, and significance was assessed by means of Student’s t-test or one-way ANOVA analysis with Bonferroni’s multiple comparison test. A p-value ≤ 0.05 was considered statistically significant.

## Results

### Multipotentiality of hSMCs

The ability of three primary lines of hSMCs to differentiate towards the osteogenic, adipogenic, and myogenic phenotypes was tested using specific media.

**Fig. 1:**
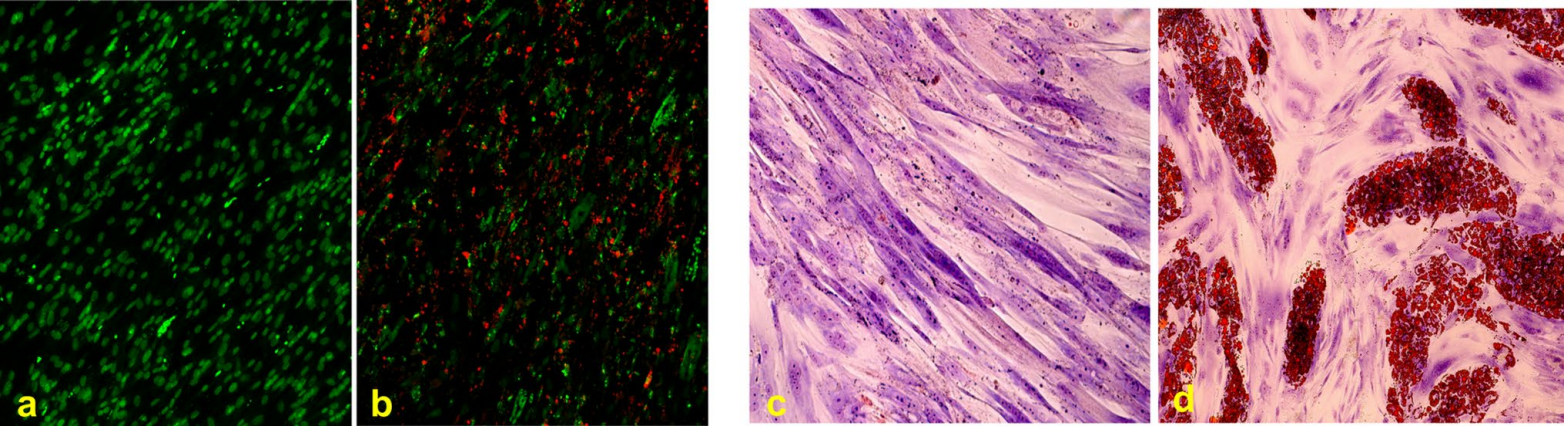
Osteogenic and adipogenic differentiation assay of hSMCs. **a**, **b** Representative images of osteogenic differentiation at 0 days (**a**) and after 21 days of induction (**b**), using appropriate medium OM, as described in “Materials and Methods”. The mineralized calcium deposits are stained in fluorescent red and nuclei counterstained with propidium iodide in conventional green color. Images acquired in epifluorescence. Original magnifications: 10x. Adipogenic differentiation assay of hSMCs. Representative images of adipogenic differentiation at 0 days (**c**) and after 14 days of induction using the appropriate medium AM (**d**), as described in “Materials and Methods”, by cytochemical staining with Oil Red O solution. Red shows the intracellular lipidic droplets, violet shows the nuclei counterstained by Toluidine Blue. Images acquired in bright field. Original magnifications: 10x

Osteogenic differentiation was assessed in hSMCs by evaluating the deposition of HA deposits, which were observed in hSMCs grown in OM for 21 days, but not in hSMCs, grown in CM (Fig. 1a-b).

The adipogenic phenotype of hSMCs was assessed by the formation of intracellular lipid vesicles using AM medium for an induction period of 14 days. In this condition, several intracellular lipid vesicles were observed in comparison to hSMCs grown in CM (control), which did not show the formation of intracellular lipid vesicles (Fig. 1c-d).

**Fig. 2:**
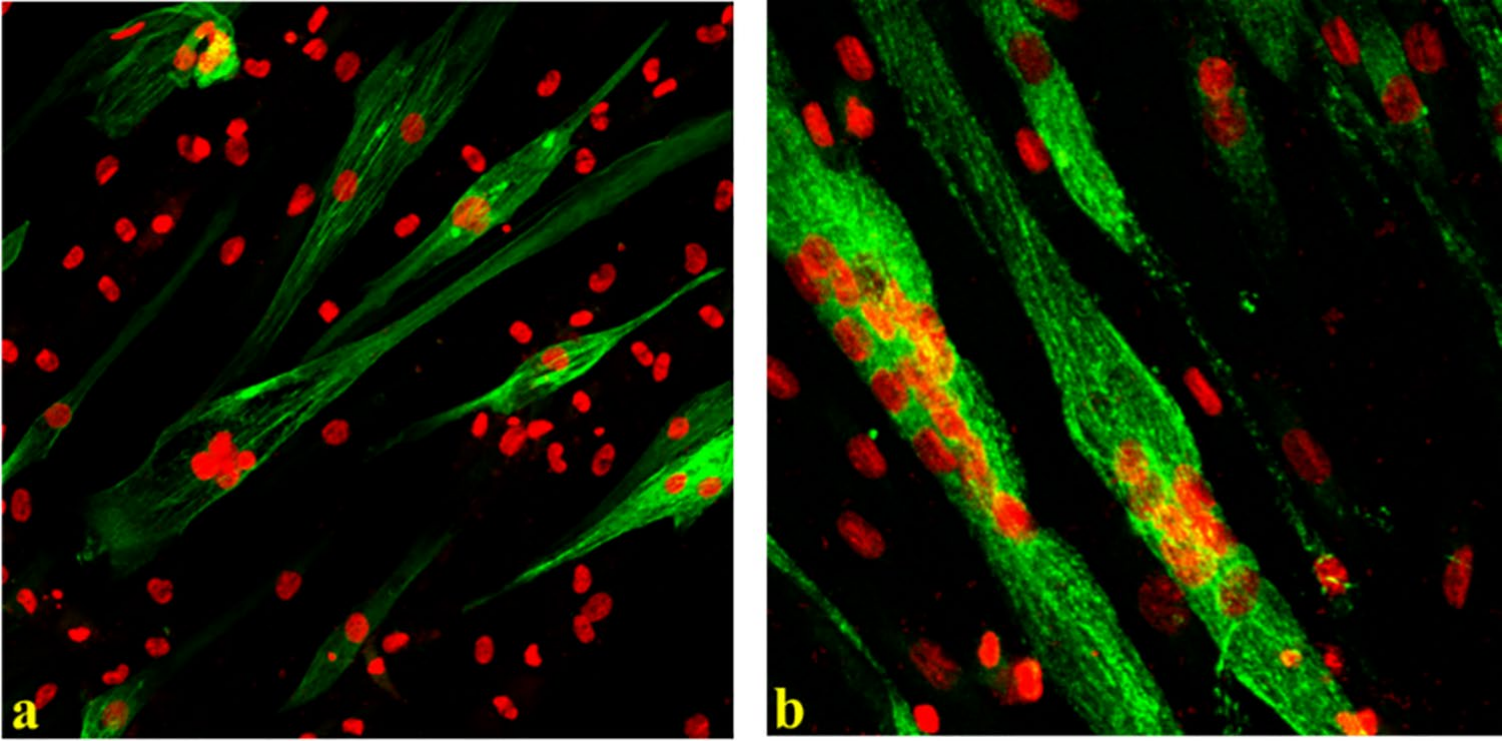
Immunofluorescence staining of MHC. hSMCs were cultured in DM for 10 days as reported in “Materials and Methods”. Fluorescence was evaluated by LSMC microscopy in conventional colors: red for MHC and green for nuclei. Original magnification: 20x (**a**) and 40x (**b**)

The ability of the hSMCs to differentiate towards the myogenic phenotype was analyzed using the appropriate DM medium for 10 days. Immunofluorescence shows that after 10 days of induction the multinucleated hSMCs express the MHC, a protein essential for skeletal muscle contraction and therefore of primary importance along with actin for muscle movement (Fig. 2).

### Effect of FGF23 Treatment on hSMCs Proliferation Process

**Fig. 3:**
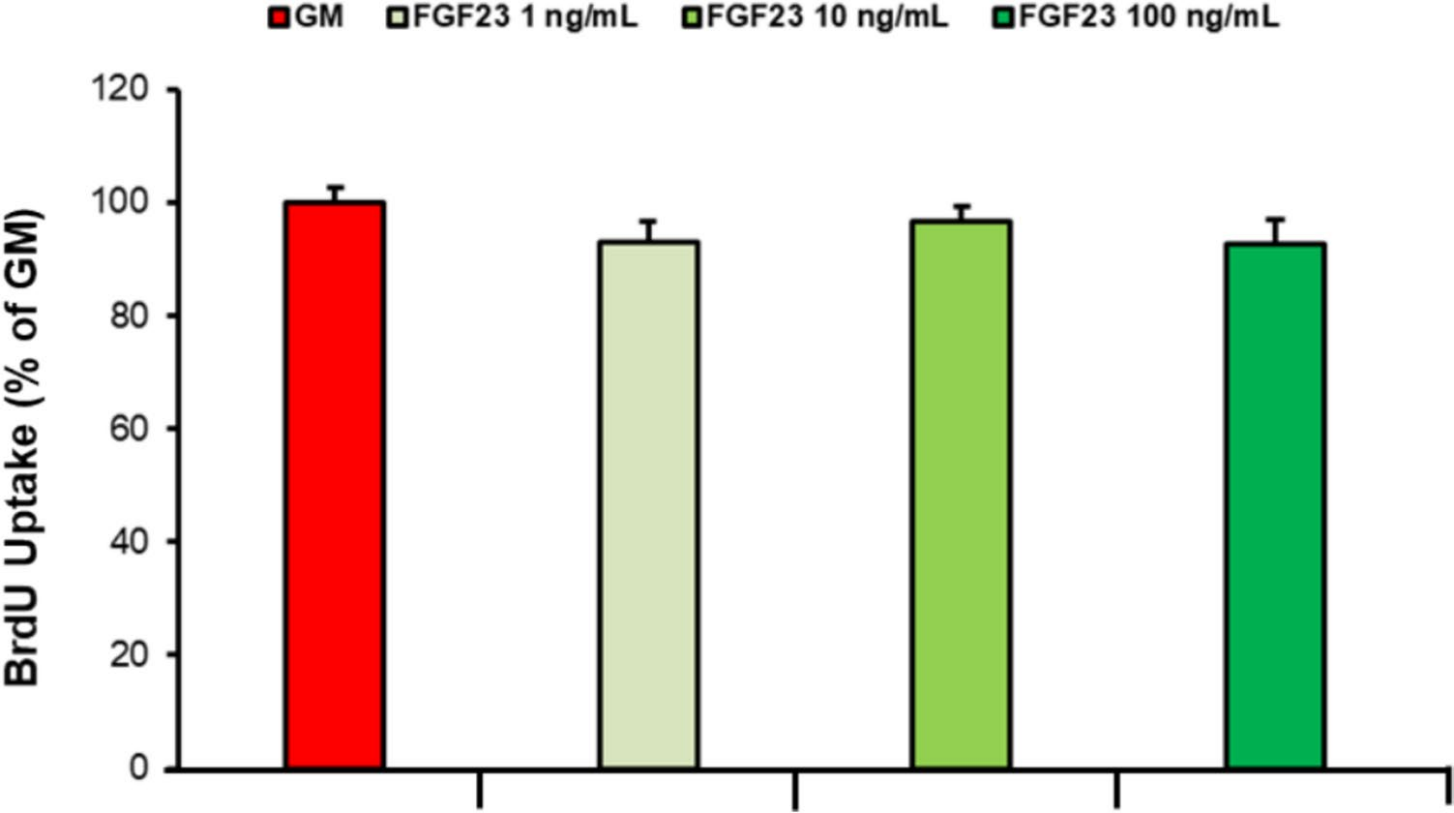
Proliferation of hSMCs treated with FGF23. hSMCs were untreated or treated with different concentrations of FGF23 (1, 10 and 100 ng/mL) for 48 h, as described in “Materials and Methods”. Proliferation was assessed with the BrdU incorporation proliferation assay and results are reported as a percentage of the respective absorbance measured in the control group. Values are the mean ± SD of three cell lines repeated in quadruplicate

Figure 3 has shown no significant differences between the hSMCs stimulated with different concentrations of FGF23 for 48 h and the untreated control, indicating that the hormone does not affect the hSMCs proliferation under the described experimental conditions.

### Effect of FGF23 Treatment on MRFs Gene Expression During hSMCs Differentiation

**Fig. 4:**
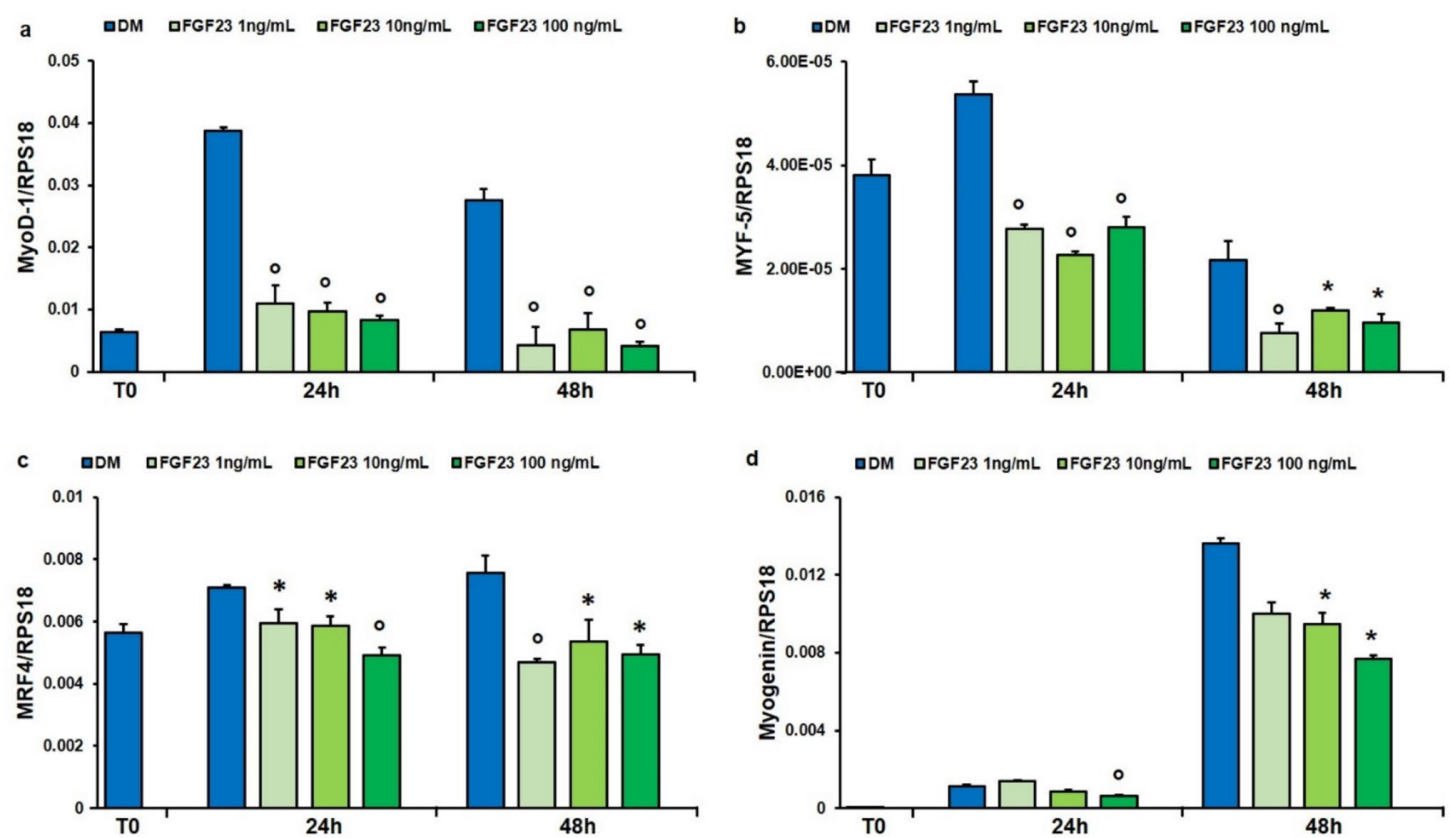
Effect of FGF23 on the expression of MRFs. qPCR analysis of the expression of *MyoD-1* (**a**), *Myf-5* (**b**), *MRF4* (**c**), *Myogenin* (**d**) in hSMCs treated with FGF23 (1, 10 and 100 ng/mL) for 24 and 48 h in DM as reported in “Materials and Methods”. Values are the mean ± SD of three independent experiments, and they are expressed as the number of mRNA molecules of the genes normalized to the housekeeping RPS18 mRNA. *p < 0.05, °*p* < 0.01 *versus* control group in DM

Several myogenic genes have been evaluated to define whether the FGF23 can affect myogenesis. hSMCs have been treated in DM in the presence or not of 1, 10, and 100 ng/mL of FGF23 for 24 and 48 h. The results showed a significant decrease in the expression of myogenic regulatory factors (MRFs) (i.e., *MyoD-1, Myf-5, MRF4*, and *Myogenin*) with all tested concentrations of FGF23 at both 24 and 48 h compared to control, with exception of *Myogenin*; in fact, expression levels analysis showed a decrease only for the highest concentration tested after 24 h and for the 10 and 100 ng/mL of FGF23 after 48 h (Fig. 4a-d).

### Effect of FGF23 Treatment on Desmin, Irisin, Myostatin and MHC Gene Expression During hSMCs Differentiation

**Fig. 5:**
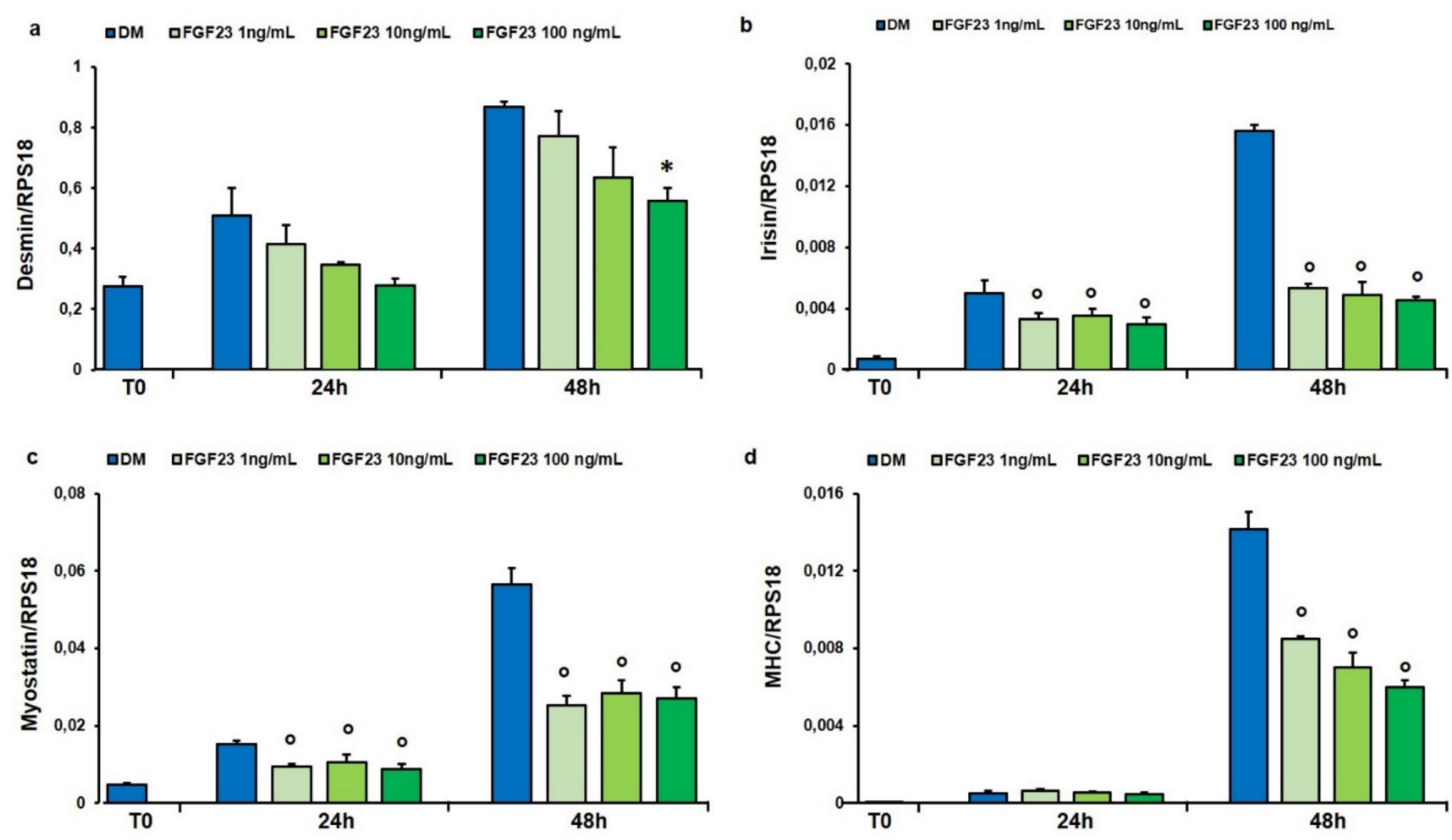
Effect of FGF23 on the expression of desmin, Irisin, myostatin and MHC. qPCR analysis of the expression of *desmin* (**a**), *irisin* (**b**), *myostatin* (**c**), *MHC* (**d**) in hSMCs treated with FGF23 (1, 10 and 100 ng/mL) for 24 and 48 h in DM as reported in “Materials and Methods”. Values are the mean ± SD of three independent experiments, and they are expressed as the number of mRNA molecules of the genes normalized to the housekeeping RPS18 mRNA. *p < 0.05, °p < 0.01 *versus* control group in DM

Desmin, irisin, and myostatin, involved in skeletal muscle development, and MHC were assessed in order to evaluate whether FGF23 could affect their expression during the early phases of myogenic differentiation. hSMCs have been treated in DM in presence or not of 1, 10, and 100 ng/mL of FGF23 for 24 and 48 h. A significant decrease in *desmin* expression was only observed at the highest FGF23 concentration after 48 h compared to control (Fig. 5a). All concentrations of FGF23 tested resulted in a significant decrease in *irisin* and *myostatin* expression at both 24 and 48 h and of *MHC* only at 48 h compared to untreated cells (Fig. 5b-d).

### Effect of FGF23 Treatment on FGFRs and KLOTHO Gene Expression During hSMCs Differentiation

**Fig. 6:**
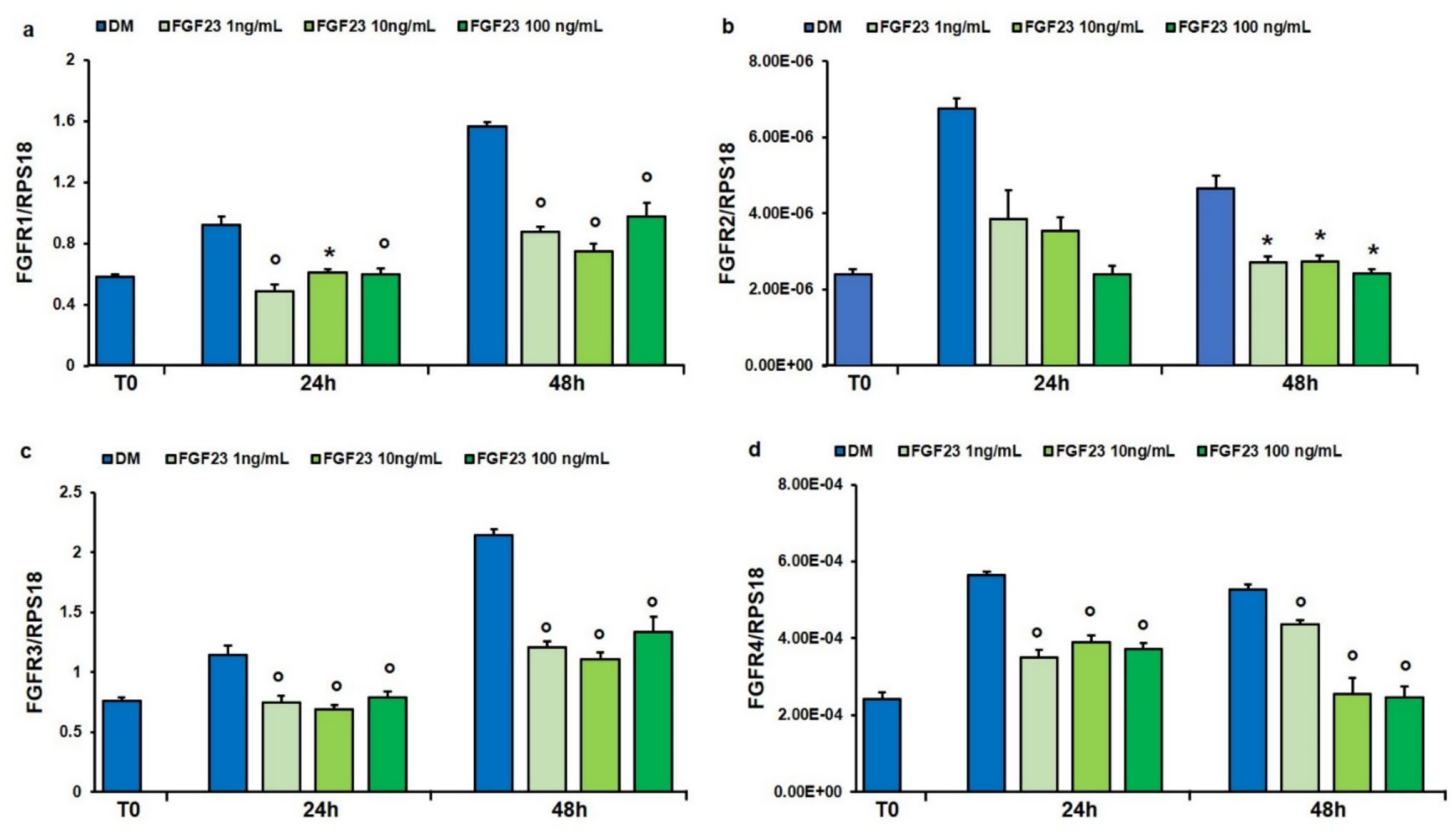
Effect of FGF23 on the expression of FGFRs. qPCR analysis of the expression of *FGFR1* (**a**), *FGFR2* (**b**), *FGFR3* (**c**), *FGFR4* (**d**) in hSMCs treated with FGF23 (1, 10 and 100 ng/mL) for 24 and 48 h in DM. Values are the mean ± SD of three independent experiments, and they are expressed as the number of mRNA molecules of the genes normalized to the housekeeping RPS18 mRNA. *p < 0.05, °*p* < 0.01 *versus* control group in DM

The effect of FGF23 on the expression of its receptors FGFRs 1–4 and the coreceptor αKLOTHO was evaluated. hSMCs have been treated in DM in presence or not of 1, 10, and 100 ng/mL of FGF23 for 24 and 48 h. The results obtained showed that all tested concentrations of FGF23 led to a significant reduction in *FGFRs 1–4* expression at all times compared to control, except *FGFR2* where a significant reduction was observed only after 48 h of treatment (Fig. 6a-d).

**Fig. 7:**
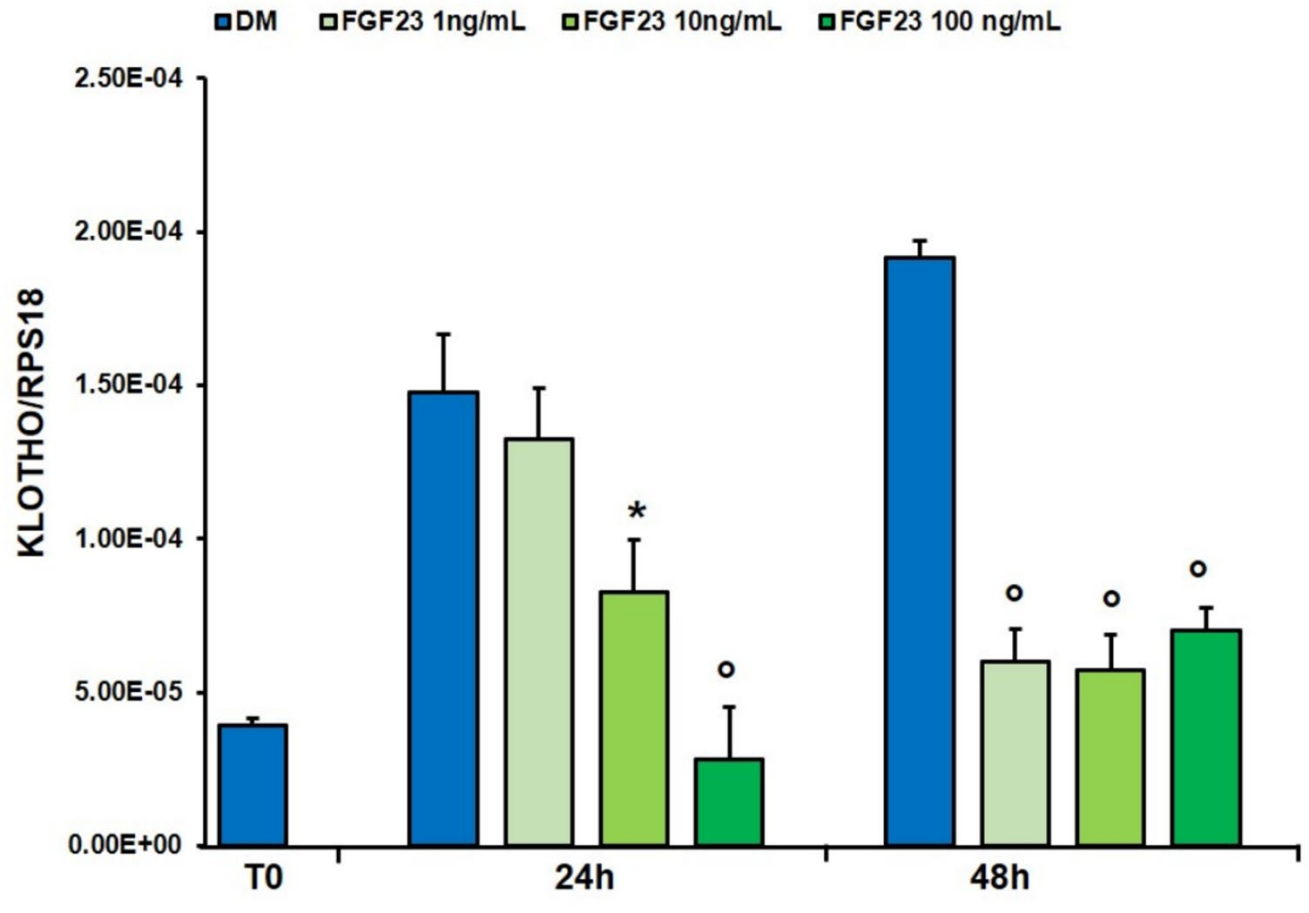
Effect of FGF23 on the expression of KLOTHO. qPCR analysis of the expression of *KLOTHO* in hSMCs treated with FGF23 (1, 10 and 100 ng/mL) for 24 and 48 h in DM as reported in “Materials and Methods”. Values are the mean ± SD of three independent experiments, and they are expressed as the number of mRNA molecules of the genes normalized to the housekeeping RPS18 mRNA. *p < 0.05, °*p* < 0.01 *versus* control group in DM

All concentrations of FGF23 resulted in a significant reduction in *KLOTHO* expression with respect to the untreated cells, except for 1 ng/mL at 24 h (Fig. 7).

## Discussion

In this study, we have shown for the first time that FGF23 in hSMCs influences myogenic differentiation but not proliferation. FGF23 belongs to the large family of FGFs, of which 22 members are known to exert paracrine or endocrine effects in bone, kidney, liver, and brain physiology [9, 26]. High FGF23 levels are responsible for the main clinical manifestations of XLH, such as hypophosphatemia, rickets, and bone mineralization defects [12]. XLH also leads to muscular disorders, such as muscle weakness, pain, reduced muscle density, peak strength, and power [17–19, 27].

The presence of muscular symptoms in XLH is not surprising as the importance of biomechanical and biochemical crosstalk by which muscle and bone, through the secretion of myokines and osteokines, respectively, communicate and influence each other has become increasingly apparent in recent years [28–30].

As very few reports take into consideration the role of FGF23 on skeletal muscle processes, our study focuses on the effects of FGF23 treatments in an in vitro model of skeletal muscle cells derived from human biopsies.

Based on previous studies, we selected three hSMC cell lines, isolated and characterized by their stem phenotype [31], in which we did not find FGF23 mRNA expression (data not shown). This finding, on the one hand, confirms that FGF23 is mainly synthesized by osteocytes and osteoblasts and, on the other, is in line with data reported in the literature that mRNAs of only 4 FGFs were found in satellite cells [32].

In this study, we used three different FGF23 concentrations (1, 10, and 100 ng/mL), which were not only in line with those used by other groups but also reflected FGF23 levels measured in the serum of patients with hypophosphatemic rickets or other diseases characterized by high FGF23 levels [33–41]. This enabled us to attempt to reproduce in vitro experimental conditions that were as similar as possible to those demonstrated in vivo.

Under our experimental conditions, FGF23 is not able to influence the proliferation of hSMCs. These data agree with the literature, where it is reported that different concentrations of FGF23 do not affect the proliferation of human or murine muscle cells [33, 35, 41]. Conditions of hypophosphataemia and moderate to severe hyperphosphataemia do not alter the proliferation rates of C2C12 murine myoblasts [42, 43]. To our knowledge, this is the first in vitro study to examine the effect of different concentrations of FGF23 on cell proliferation in the presence of low phosphate levels. Therefore, we hypothesize that there is no activation of pathways affecting cell proliferation under these experimental conditions.

However, FGF23 affects the process of myogenic differentiation, a complicated and finely regulated biological process involving Myf-5, MyoD-1, myogenin, and MRF4, which make up the family of MRFs [44, 45]. MRFs are muscle-specific proteins that guide progenitor cells to establish the skeletal muscle phenotype [44, 45]. Besides these, other important factors involved in the myogenic differentiation process are desmin, irisin, and myostatin, which are involved in skeletal muscle development, and MHC, a protein essential for skeletal muscle contraction and, therefore, of primary importance along with actin for muscle movement [46–49]. FGF23 under our experimental conditions resulted in a significant decrease in the expression of these genes in hSMCs.

Although some works report no effect of FGF23 on murine C2C12 muscle lines, some data support our results [29, 31]. In fact, other members of the FGFs family (such as FGF2, 9, 16, 20) cause an inhibition of the differentiation of C2C12 cells, human skeletal muscle cells, and myoblasts [35, 50, 51]. Furthermore, FGF23 has been shown to induce the senescence of mesenchymal stem cells derived from skeletal muscle, which, although having different properties from satellite cells, support muscle differentiation and regeneration [41].

FGF23 exerts its actions through its FGFRs receptors with the presence of the α-KLOTHO coreceptor [5]. We confirmed the presence of FGFRs and α-KLOTHO in GM-cultured hSMCs, as reported in the literature [33, 52, 53]. In our experimental conditions, FGF23 resulted in a significant reduction in the levels of the 4 *FGFRs* and the *α-KLOTHO* coreceptor compared to untreated hSMCs. We hypothesize that this is due to the establishment of a negative feedback mechanism that aims to reduce the effects induced by high FGF23 levels. Latic et al. also showed that in the kidney of the Hyp mice, the animal model of hypophosphatemia, the abundance of the α-Klotho protein decreases by half compared to wild-type controls [54].

All these collected data suggest that FGF23 is indeed involved in the muscle disorders that occur in XLH. Indeed, in 2018 in Europe and the USA a fully human immunoglobulin IgG1 monoclonal antibody directed against FGF23 (Burosumab), which prevents FGF23 binding to FGFRs, was approved for the treatment of XLH [55]. In preclinical studies performed on Hyp mice, Burosumab resulted in improved hypophosphatemia, 1,25(OH)_2_D levels, and it was also observed that it was able to improve muscle strength. Aono et al. demonstrated that administration of anti-FGF23 antibodies in adult Hyp mice increased not only muscle strength but also the frequency of spontaneous movements [56]. In patients with XLH, the administration of Burosumab resulted in a better perception of their physical performance [57].

Although further studies are needed, we can speculate that the improvement in muscle function observed in patients with XLH after treatment with Burosumab is due to increased serum phosphate levels and reduced FGF23 action in skeletal muscle. In particular, patients with hypophosphatemic rickets exhibit qualitative and quantitative muscle deficits, including reduced muscle density and volume, as well as altered contractions and elongation-shortening cycles [17]. Our results could explain how Burosumab positively affects on skeletal muscle of XLH patients reducing FGF23 action, since we have reported that FGF23 is able to impair myogenic differentiation reducing the expression of MRFs. The regulation of MRFs expression is fundamental, as MRFs are involved in the activation of satellite cells and myogenic differentiation, as well as being closely connected with signaling pathways involved in adaptation, muscle development, and regeneration [58]. Therefore, a reduction in MRFs expression levels may be associated with structural and functional deficits in skeletal muscle [59]. Therefore, if it is caused by the presence of FGF23, a reduction of FGF23 action, as reported under treatment with Burosumab, could be one of the positive effects of this treatment, since it is able to block FGF23 activity.

Despite, this first study on the effects of FGF23 on muscle physiology presents some limitations such as the small number of tested hSMCs and the fact that we do not know the FGF23 levels of the three donors, an aspect that could be interesting to evaluate the effects of FGF23 among patients which present different circulating levels of FGF23 [60].

However, the reported results have shown the in vitro effects of FGF23 on the proliferation and myogenic differentiation of hSMCs. Although such treatments do not affect cell proliferation at the experimental tested condition, it induces a decrease in *MRFs*, *desmin*, *irisin*, *myostatin*, *MHC*, *FGFRs*, and *α-KLOTHO* gene expression during differentiation, indicating the possible involvement of FGF23 in muscular dysfunction characterizing hypophosphatemic rickets and the possible presence of negative feedback providing protection against high FGF23 levels.

Further studies, using a human in vitro model for clarifying these effects at the molecular and cellular levels, are needed for a better comprehension of XLH muscular impairment and, consequently, of its management. A deeper knowledge of the precise mechanism in human cellular models may lead to the development of new therapeutic strategies to prevent and treat this disease. These results will make possible to evaluate whether the muscular symptoms in XLH are related to FGF23 excess in skeletal muscle.
